# Potential antimicrobial properties of the *Ulva lactuca* extract against methicillin-resistant *Staphylococcus aureus*-infected wounds: A review

**DOI:** 10.14202/vetworld.2021.1116-1123

**Published:** 2021-05-08

**Authors:** Nadya Fianny Ardita, Lenny Mithasari, Daris Untoro, Siti Isrina Oktavia Salasia

**Affiliations:** 1 Faculty of Veterinary Medicine, Universitas Gadjah Mada, Yogyakarta, Indonesia; 2 Department of Clinical Pathology, Faculty of Veterinary Medicine, Universitas Gadjah Mada, Yogyakarta, Indonesia

**Keywords:** antibacterial, methicillin-resistant *Staphylococcus aureus*, *Ulva lactuca*, wound infection

## Abstract

Methicillin-resistant *Staphylococcus aureus* (MRSA), currently a major problem in hospitals worldwide, is one of the most common causes of nosocomial disease through surgical wound infection. MRSA-infected wounds have very low recovery rates and have become more problematic as some antibiotics are not effective against MRSA. Several antimicrobial and anti-inflammatory agents of green algae (*Ulva lactuca*) in the form of alkaloids, triterpenoids, steroids, saponins, and flavonoids have the potential to accelerate the wound healing process following MRSA wound infection. Various active compounds contained in the *U. lactuca* extract are thought to have multiple antibacterial and anti-inflammatory properties that can overcome the MRSA antimicrobial resistance and accelerate tissue growth in the wound healing process. This review aims to describe the potential of *Ulva lactuca* extract against MRSA-infected wound healing.

## Introduction

Nosocomial infections are infections acquired from the hospital during medical treatment. About 15% of the hospitalized patients acquire nosocomial infections [1]. Worldwide statistics show that nosocomial infections occur in 7% of all disease incidences in developed countries and in 10% of disease incidences in developing countries. Surgical site infection (SSI) is considered one of the most common routes of nosocomial disease [2]. Nosocomial pathogens include bacteria, viruses, and fungal parasites. *Staphylococcus aureus* is often considered an agent of nosocomial infection [3]. S. aureus, from which some strains have evolved into Methicillin-resistant *S. aureus* (MRSA), is a bacteria that are resistant to various kinds of antibiotics; it is also known as a *superbug* (multi-resistant bacteria) [4]. MRSA is currently recognized as a significant problem in hospitals worldwide [3,5]. *S*. *aureus* resistant strains can interfere with the wound healing process, which causes prolonged wound healing, and lead to increased mortality and morbidity [6-8]. Until now, there is no anti-MRSA agent that is accepted globally.

*Ulva lactuca*, also known as green algae (sea lettuce), is classified as macroalgae in the phylum Chlorophyta [9]. *U. lactuca* contains secondary metabolites such as alkaloids, triterpenoids, steroids, saponins, phenolic compounds, and flavonoids [10,11]; these active compounds have the potential to accelerate wound healing of nosocomial wound infections due to their antibacterial, anti-inflammatory, antioxidant, and anticoagulant activities [12]. With various active compounds in the *U. lactuca* extract, it is thought to be effective against multi-resistant bacteria. This extract also acts through multiple antimicrobial mechanisms to penetrate bacterial resistance barriers [13].

Information on the potential of *U. lactuca* as an antibacterial and anti-inflammatory agent in wound healing is still limited. This review addresses what is known about the potency of *U. lactuca* as a source of anti-MRSA agents.

## SSI and Inflammation

SSI increases morbidity and mortality, thereby increasing health cost burdens and lowering patients’ quality of life, as reported in European countries [14]. As summarized by Mawalla *et al*. [5], SSI rates have been reported to range from 2.5% to 41.9% globally.

According to the European Center for Disease Prevention and Control, SSIs are divided into superficial, deep, and organ/cavity infections (Table-1) [4,5,15]. Superficial infection indicates local wound symptoms, such as sero-sanguineous-to-purulent discharge from the wound or accumulation of pus associated with swelling, redness, heat, and pain. Deep infection is related to symptoms such as systemic sepsis, including fever, tachypnea, and left shift leukocytosis. Radiological or ultrasound examination is required to determine organ/cavity infections [5,15,16]. A prospective cross-sectional study has been reported by Mawalla *et al*. [5] involving patients who underwent major surgery. SSI was detected in 26.0% patients, of whom 86.2% had superficial SSI and 13.8% had deep SSI. Among patients with clinical SSI, 86.2% were positive for aerobic culture [5].

**Table-1:** Surgical site infection.

Type of infection	Part involved	Time of infection	Clinical symptoms
Superficial	Skin, subcutaneous tissue	30 days after surgery	Local wound symptoms such as serosanguineous to purulent discharge from the wound or accumulation of pus associated with swelling, redness, heat, and pain
Deep	Soft-tissue (fascia, muscle layer)	90 days after surgery	Symptoms of systemic sepsis include fever, tachypnea and left shift leukocytosis
Organs/cavities	Internal organs, body cavities, bones	90 days after surgery	Radiological or ultrasound examination is required

Source: World Health Organization [4]; Mawalla *et al.* [5]; Griffon and Hamaide [15]

The most common infectious agents found in surgical wounds include *S. aureus, coagulase-negative Staphylococci, Enterococcus* species, and *Escherichia coli*. At present, there has also been an increase in the incidence of SSI caused by antibiotic-resistant pathogens, such as MRSA [15]. Mawalla *et al*. [5] reported that *S. aureus* was the predominant (28.6%) SSI organism, as 18.8% of the SSI cases reported in major surgery patients were infected with MRSA.

Inflammation is a defense mechanism against pathogens and other causes of tissue damage [17]. The molecular mechanisms of inflammation are fairly complex and begin with the recognition of specific molecular patterns associated with cellular infections or injury. The entire inflammatory response is mediated by several key regulators involved in the selective expression of pro-inflammatory molecules [18]. This complex response involves leukocytes, such as macrophages, neutrophils, and lymphocytes, which are also known as inflammatory cells [17]. Various chemical mediators from the circulatory system, inflammatory cells, and injured tissues actively contribute to and adjust the inflammatory response. The chemical mediators released include (1) vasoactive amines (e.g., histamine and serotonin), (2) peptides (e.g., bradykinin), and (3) eicosanoids (e.g., thromboxane, leukotrienes, and prostaglandins) [19].

The damage-associated molecular patterns and pathogen-associated molecular patterns of invading bacteria, fungi, or viruses are the main mediators of cell injury and microbial invasion, respectively. The molecular patterns are detected by receptors called pattern recognition receptors (PRRs). PRRs, which give rise to direct immune response, are constitutively expressed on host macrophages, monocytes, dendritic cells, neutrophils, and epithelial cells. The first inflammatory leukocytes to be recruited to the wound site are neutrophils. Under the influence of chemokines, there is an increase in the expression of adhesion molecules (ICAM, VCAM1, and e-selectin) on vascular endothelial cells, leading to the adherence of neutrophils to the vessel wall, followed by extravasation and migration along the chemokine gradient. This neutrophil migration is accompanied by phagocytosis, killing of ingested bacteria, release of antimicrobial cationic proteinases and peptides, and production of tumor necrosis factor (TNF)-a, interleukin (IL)-1b, and IL-6, which maintain the inflammatory state [20].

## *Staphylococcus aureus* and Multi-resistant Superbug

*S, aureus* is an invasive bacterium that can infect both healthy and unhealthy individuals in hospitals and through infected communities. *S. aureus* is also well adapted to human hosts. It can cause bacteremia, septicemia, soft-tissue infections, endocarditis, osteomyelitis, pneumonia, and meningitis [21]. *S. aureus* infection causes significant morbidity in some infected patients and a mortality rate that may approach 40% [22].

The nasal carriage of multi-resistant S. aureus seems to be the origin for the development of staphylococcal infections in patients with post-surgical wounds [21-24]. *S. aureus* is commonly adherent to the skin of atopic-dermatitis patients, and prolonged infection may exacerbate the clinical condition [25,26]. Staphylococcal enterotoxin Y may accelerate localized inflammation through skin-resident T-cell activation, thereby facilitating the pathogenesis of *S. aureus* infection in patients with disrupted epithelial barriers [26]. The persistent carriage of strains harboring these virulence determinants may increase the risk for subsequent invasive infections in carriers [21-23,25,26].

*S. aureus* expresses a variety of virulence factors, including toxins (hemolysin and leukocidins), surface immune-evasive factors (capsules and protein A), and enzymes that promote tissue invasion (hyaluronidase enzymes) [24]. Microorganisms that can develop resistance to antimicrobials are called superbugs [4]. Antimicrobial resistance (AMR) occurs when microorganisms (such as bacteria, fungi, viruses, and parasites) adapt when exposed to antimicrobial drugs (such as antibiotic, antifungal, antiviral, antimalarial, and anthelmintic drugs). “Superbug” is a term used to describe the evolution of bacterial species resistant to antibiotics. Antibiotic resistance by superbugs causes economic losses by increasing the duration of infections, increasing the cost of treatment, and decreasing the success of surgical treatment due to nosocomial infections [27]. AMR threatens the effective prevention and treatment of a growing range of infections caused by bacteria, parasites, viruses, and fungi. As a result, drugs become ineffective and the infections remain in the body, thereby increasing the risk of transmission to others [4].

## Resistance Mechanism

The use of large quantities of antimicrobials over a long period of time can provide selection pressures that support the evolution of resistant strains. Resistant strains can spread horizontally and cause the spread of the virus in the environment [27-29]. From an evolutionary perspective, bacteria use two main genetic strategies to adapt to antibiotic attack: (1) Gene mutations that are often associated with the mechanism of action of the antibiotic compounds and (2) acquisition of foreign DNA coding for determinants of resistance through horizontal gene transfer (HGT) [27,29].

Gene mutations can accelerate the resistance to antimicrobial molecules. Once resistant mutants emerge, antibiotics eliminate the susceptible population, and resistant bacteria predominate. In general, mutations resulting in AMR alter the action of antibiotics through one of the following mechanisms: (1) Modification of the antimicrobial target by decreasing bacterial affinity for the drug, (2) reducing drug uptake, (3) activating the excretion mechanism to excrete harmful molecules, or (4) global change in important metabolic pathways through modulation of regulatory networks. Therefore, raising microbial resistance due to mutational changes can be varied and complex [27,29].

HGT is one of the most important drivers of bacterial evolution and is often responsible for developing AMR. Classically, bacteria acquire external genetic material through three main strategies, (1) transformation (fusion of bare DNA), (2) transduction, and (3) conjugation. The emergence of resistance in a hospital setting often involves conjugation, a highly efficient gene transfer method that involves cell-to-cell contact, and is most likely to occur at high levels in the human gastrointestinal tract under antibiotic treatment [27,29].

The main antibiotic resistance mechanisms in superbugs can be briefly described as follows: (1) Production of hydrolysis products such as β-lactams by various β-lactamase enzymes (Class A to D β-lactamases), (2) changes in penicillin-binding proteins to prevent β-lactam action, (3) changes in the structure and amount of porin protein, resulting in decreased permeability to antibiotics through the outer membrane of bacterial cells, and (4) efflux pump activity, which further reduces the antibiotic concentration in bacterial cells [28,29].

## MRSA Infection

Resistance to *S. aureus* first emerged in the 1940s, mediated by the bacterial β-lactamase gene [24]. Hospital-acquired infections are still a major cause of morbidity and mortality worldwide. MRSA has been identified as one of the most frequently infecting nosocomial pathogens, accounting for about 25% of over 2 million nosocomial infections ranging from mild skin infections to septicemia [30,31]. Vancomycin is the last choice of antibiotics that can inhibit the growth of MRSA bacteria. The emergence of resistance to vancomycin is the most feared genetic adaptation in *S. aureus* till date [24]. Bacteria that are completely resistant to vancomycin are also considered as superbugs [28,32].

In general, risk factors that predispose patients to postsurgical MRSA infections include: (1) Duration of surgery or anesthesia, which may explain the increased risk of infection associated with simultaneous bilateral orthopedic procedures, and (2) surgical implant placement, in which the surgical plate acts as a nidus for infection and a site for biofilm formation, which can help bacteria avoid the immune system and antibiotics [16].

The body’s main defense against *S. aureus* infection is the neutrophil response. When *S. aureus* enters the skin, neutrophils and macrophages migrate to the infection site. *S. aureus* avoids this response and increases the number of skin and soft-tissue infections in many ways, including blocking leukocyte chemotaxis, confiscating host antibodies, hiding from detection through polysaccharide capsules or biofilm formation, and fighting damage after ingestion by phagocytes. Several virulence factors appear to contribute to infection, including Panton–Valentine leukocidin (PVL), a-hemolysin (also called alpha-toxin), phenol-soluble modulin (PSM), arginine catabolic mobile element (ACME), and regulatory loci referred to as Agr. PVL causes lysis of white blood cells. In a meta-analysis of several studies, the presence of PVL was clearly associated with abscesses and furuncles at a ratio of 10.5 (95% CI, 7.4-14.9). a-hemolysin is a toxin that can form pores in various human cells and cause cell lysis. In skin infections, a-hemolysin also contributes to keratinocyte penetration. PSM and its proteolytic products facilitate MRSA colonization and lyse human cells, including neutrophils and erythrocytes. The s*pe*G gene in the ACME locus also increases resistance to polyamines produced by the skin, thus leading to the possibility of a selective advantage during colonization and bacterial infection of the skin. The *Agr* locus regulates the release of virulence factors such as PVL, a-hemolysin, and ACME from MRSA bacteria [30-33].

In a study conducted by El-Gayar *et al*. [34], macroscopic and microscopic results were obtained for wounds on the skin of rats infected with MRSA. Biopsies were taken from rats of MRSA-infected and uninfected group immediately after fasting for different time intervals, fixed in 10% formalin salt solution for 24 h, and stained with hematoxylin and eosin for examination under a microscope. Macroscopically, wounds without MRSA infection did not show any pus, whereas wounds with MRSA infection appeared wet (oozing) and pus formation began after 5-11 days. Microscopically, wounds without MRSA infection showed no histopathological changes, whereas wounds with MRSA infection showed necrosis of the epidermis and dermis on day 3 and ulceration of the epidermis and dermis on day 9.

## Benefits of Green Algae (*U. lactuca*)

U. lactuca is a green algae, commonly known as sea lettuce, belongs to the phylum Chlorophyta. This sessile algae is able to grow and float freely [35,36]. U. lactuca reproduces through sexual reproduction, and also through thallus fragmentation, although rare. U. lactuca is a polymorphic species with a morphology that depends on salinity or symbiosis with bacteria [9]. *U. lactuca* is considered a widespread therapeutic herbal source for some oriental remedies of some noxious diseases [36,37].

As reported by Amin [10] and Habbu *et al*. [11], *U. lactuca* contains secondary metabolites in the form of alkaloids, triterpenoids, steroids, saponins, phenolic compounds, and flavonoids, which are useful as antimicrobial agents (Table-2) [13,37-42]. The benefits of *U. lactuca* can be seen in Table-3 [42-47], including antioxidant, antimicrobial, antiviral, antihyperlipidemic, antitumor, anti-inflammatory, antibiofilm, and anticoagulant activities [13,43-48]. The antibacterial activity of the *U. lactuca* extract against *S. aureus* is detailed in Table-4 [10,11,49-51]. The antibacterial activity of seaweeds is generally assayed through extraction in various organic solvents, such as acetone, methanol-toluene, ether, or chloroform-ethanol [52]. The use of organic solvents generally leads to higher efficiency in extracting compounds for antimicrobial activity [53].

**Table-2:** Pharmacological activity of the active compound of *U. lactuca.*

Chemical compounds	Pharmacological activity	References
Saponin	Accelerates the wound healing process and inhibits inflammatory reactions in wounds during the initial phase	[38]
Steroid	Antimicrobial agent	[39]
Triterpenoid	As an anti-tumor, antibacterial, anti-inflammatory, and anti-hyperglycemic	[40]
Alkaloid	Inhibits the growth of Gram-positive bacteria	[41]
Flavonoid	Has antioxidant properties that can reduce cell necrosis and wound tissue damage due to oxidation by repairing damaged blood vessels and reducing lipid peroxidation, has antibacterial activity by inhibiting bacterial growth and is able to maintain wound contraction and increase the rate of epithelialization	[13,41]
Tannin	Antimicrobial agent	[39]
Fenol	Antioxidants	[42]

*U. lactuca=Ulva lactuca*

**Table-3:** Benefits of *U. lactuca.*

Efficacy	Treatment	Method	Result	References
Anti-inflammatory	Acute inflammatory leg edema (Wistar rats)	The extract was injected into the legs; Foot thickness was measured before and after injection at 0, 1, 3, and 6 h	*U. lactuca* extract had a significant anti-inflammatory effect (48%-51%) at 3 h after treatment compared to diclofenac-sodium as a positive control showing an anti-inflammatory effect of 52%	[43]
Anti-inflammatory antioxidants	Rheumatism (Wistar rats)	Extract was given orally; for 1, 2, and 3 weeks ankle circumference was measured	Treatment of rheumatic rats with *U. lactuca* extract induced significant improvement at 1^st^ and 2^nd^ weeks. *U. lactuca* extract had strong anti-inflammatory effects and antioxidant properties	[44]
Anti-bacteria	-	The extracts were tested on an antibiotic disc and the inhibition zone diameter was measured	Antibacterial activity revealed against pathogens *S. aureus*, *K. pneumonia, P. vulgaris* and *B. subtilis*	[45]
Anti-biofilm	-	The effect of *U. lactuca* extract and ZnO NP fabrication of *U. lactuca* on biofilms was assessed by crystal violet (CV) assay	*U. lactuca* extract showed biofilm reduction against *B. licheniformis* (85%), *B. pumilus* (79%) *E. coli* (82%), *P. vulgaris* (80%), and *U. lactuca*-fabricated ZnO NPs showed a reduction in *B. licheniformis* (90%), *B. pumilus* (89%), *E. coli* (90%), and *P. vulgaris* (91%)	[46]
Anticoagulants	-	Activated partial thromboplastin time, prothrombin time and thrombin time were measured by the Labtest kit in a Cascade-M coa-gulometer	There is anticoagulant activity of sulfated polysaccharides isolated from *U. lactuca*	[47]

*S. aureus=Staphylococcus aureus, U. lactuca=Ulva lactuca, E. coli=Escherichia coli*

**Table-4:** *U. lactuca* extraction results based on solvents and antibacterial activity against *S. aureus.*

Extract/solvent	Secondary Metabolites							Zone of inhibition against *S. aureus* (mm/mg)	References
	Saponin	Steroid	Terpenoid	Alkaloid	Flavonoid	Tannin	Phenol		
Acetone	+	+	±	±	+	±	+	26	[10,49]
Benzene	+	-	-	+	+	-	+	26	[10]
Chloro-form	±	±	±	±	±	±	+	11,26	[10,11,49,50]
Aqueous	+	+	+	+	+	-	+	26	[10]
Ethanol	+	+	+	+	+	-	+	26	[10]
Ethyl acetate	±	±	±	±	+	±	+	26	[10,11,49]
Hexane	+	±	±	±	+	-	+	26	[10,49]
Methanol	±	+	±	±	+	±	+	13, 26	[10,49,51]
Petro-leum ether	+	-	-	+	+	-	+	26	[10]

*S. aureus=Staphylococcus aureus, U. lactuca=Ulva lactuca*

## Antibacterial Activity of *U. lactuca* Against MRSA

The *U. lactuca* extract has been tested *in vitro* against several Gram-positive and Gram-negative bacteria as well as some fungi [35,38,52,54]. According to Abdel-Khaliq *et al*. [55], *U. lactuca* showed an average inhibition zone of 22.0±0.8 mm against Gram-positive bacteria *S. aureus*, which is higher than that showed by *Ulva intestinalis* (20.1±0.4 mm). The inhibition zone of *U. lactuca* was almost equivalent to the antibiotic ampicillin, which showed an inhibition zone of 28.3±0.1 mm.

Kolanjinathan and Stella [52] reported that the *U. lactuca* methanol extract (5.0 mg/mL) showed an average inhibition zone of 13±0.6 mm against the Gram-positive bacilli *S. aureus*, with a minimum inhibitory concentration (MIC) value of 2.5 mg/mL. Talreja [35] evaluated the methanolic extract of *U. lactuca* against resistant strains of human pathogenic bacteria species. Lauric, palmitic, linolenic, oleic, stearic, and myristic acids are known to be potential antibacterial and antifungal agents [56], and these compounds in the methanolic extract of *U. lactuca* may confer their antimicrobial activity against resistant strains [35]. The crude extract of *U. lactuca* also showed significantly stronger antimicrobial activity (zone of inhibition, 8.2 mm) against MRSA infections than that shown by the standard drug streptomycin (4.6 mm). Thus, crude antimicrobial activity was significant in the case of MRSA [35,57].

Kim *et al*. [58] reported that the *U. lactuca* ethyl-ether extract was able to suppress bacteria, including MRSA with MIC MRSA CCARM3561 (12.5 mg/mL), MRSA CCARM3115 (12.5 mg/mL), and MRSA CCARM3089 (50 mg/mL). Another study also showed that there is a significant antibacterial activity increase from *U. lactuca* specifically against MRSA when they are harvested at lunar Phase III [59].

## Potential of *U. lactuca* as an Anti-inflammatory Agent in Wound Healing

The inflammatory response is an essential aspect of the tissue response to harmful infections. A prolonged inflammatory process leads to chronic disease, which can eventually lead to tissue damage [17]. Several compounds isolated from marine algae possess varied chemical structures that have potent anti-inflammatory and antioxidant activities [37,60-63]. These marine algae compounds may affect multiple targets in the immune and inflammatory systems that influence disease progression [43]. *U. lactuca* contains active compounds such as saponins, terpenoids, and flavonoids that function as anti-inflammatory agents [38-40,64].

Mezdour *et al*. [43] reported that the *U. lactuca* extract that was subcutaneously injected into rats with leg edema produced a significant anti-inflammatory effect (48-51%) at 3 h after treatment, equivalent to the positive control diclofenac-sodium (52% anti-inflammatory effect). The *U. lactuca* lectin and polysaccharide extracts exerted anti-inflammatory activities by suppressing the LPS-stimulated production of TNF-α and NO in the RAW 264.7 cells treated with LPS [43,65]. Awad [66] reported the topical anti-inflammatory activity of *U. lactuca* using mouse ear edema as an experimental model. *U. lactuca* was shown to contain the anti-inflammatory compound 3‐O‐β#x2010;D glucopyranosyl‐stigmasta‐5,25‐dien. Devaki *et al*. [67] also showed that sulfated polysaccharides from *U. lactuca* could prevent the oxidative stress induced by D-galactosamine intoxication.

A similar study was also conducted by de Araújo *et al*. [68], who showed that the *U. lactuca* extract has an anti-vascular anti-inflammatory effect through the targeting of bradykinin, which is one of the chemical factors that stimulate the inflammatory process. The anti-inflammatory activity of the *U. lactuca* extract was shown to accelerate the wound healing process, as evidenced in the study of Premarathna *et al*. [69]. These authors reported that after 7 days of treatment with the *U. lactuca* extract, rats with excised wounds showed 61.67% wound healing compared with the control group without treatment (52.76%). Based on this description, *U. lactuca* has potential in accelerated wound healing because of its activity as an anti-inflammatory agent.

## Conclusion

MRSA is the primary strain of antimicrobial-resistant bacteria in hospitals. It is the leading cause of nosocomial infection and secondary infection in postoperated patients. As of yet, there is no anti-MRSA agent that is accepted globally. U. lactuca contains active compounds that have the potential to accelerate wound healing of nosocomial wound infections due to its antibacterial, anti-inflammatory, antioxidant, and anticoagulant activities. With various active compounds contained in the U. lactuca extract, it may demonstrate efficacy against multi-resistant bacteria. However, information on the potential of U. lactuca as an antibacterial and anti-inflammatory agent in wound healing is still limited. This review describes the potential of *U. lactuca* as a source of anti-MRSA agent that might be used to treat common SSIs.

## Authors’ Contributions

SIOS conceived, wrote, and revised the manuscript. NFA, LM, and DU wrote, prepared, and revised the manuscript. All authors read and approved the final manuscript.
